# When less is more: A minimally invasive, intrathoracic approach to surgical stabilization of rib fractures

**DOI:** 10.1016/j.tcr.2021.100452

**Published:** 2021-03-11

**Authors:** Zachary M. Bauman, Ryan Beard, Samuel Cemaj

**Affiliations:** Division of Trauma, Emergency General Surgery and Critical Care Surgery, Department of Surgery, University of Nebraska Medical Center, Omaha, NE 68198, USA

**Keywords:** Surgical stabilization rib fractures, Thoracoscopy, Rib fixation, Video assisted thoracic surgery (VATS), Rib fractures

## Abstract

Rib fractures are a common and serious injury in both blunt and penetrating trauma to the thorax. The morbidity and mortality associated with these fractures is often due to impaired respiratory mechanics, leading to a number of pulmonary complications and lengthy hospitalization. Surgical stabilization of rib fractures has become an increasingly popular treatment option within the trauma community, but the definitive role in management is still being established. The development of new minimally invasive techniques offers a promising future into rib fixation and its role in the treatment algorithm. Here we present a 48-year-old male who sustained four right sided rib fractures after a ground level fall and subsequently endured poor pain control and breathing mechanics requiring surgical stabilization of his rib fractures. We introduce a new minimally invasive technique for stabilization of rib fractures using an intrathoracic thoracoscopic approach. Given the patient's dramatic improvement in pain and respiratory function after surgery, we are excited about the future of this technique and all its potential.

## Introduction

Rib fractures are a common injury in both blunt and penetrating trauma. It is estimated that 10% of all traumatically injured patients sustain rib fractures, which can lead to numerous pulmonary complications [1,2]. Furthermore, the severe pain associated with these fractures is a major cause of morbidity and mortality resulting in over 450,000 hospitalizations annually in the US [3,4].

The advent of surgical stabilization of rib fractures (SSRF) has become an increasingly popular treatment option in recent years, but its formal role in rib fracture management is still being aggressively studied. The development of new minimally invasive SSRF techniques offers a promising future aimed at minimizing procedural pain and maximizing improvements in respiratory status. Thoracoscopic-assisted intrathoracic SSRF is a new minimally invasive technique designed to offer the biomechanical advantages from rib fracture stabilization while minimizing the invasiveness of the procedure. Below we present a case of SSRF using this technique, achieving very desirable results.

## Case presentation

**Fig. 1:**
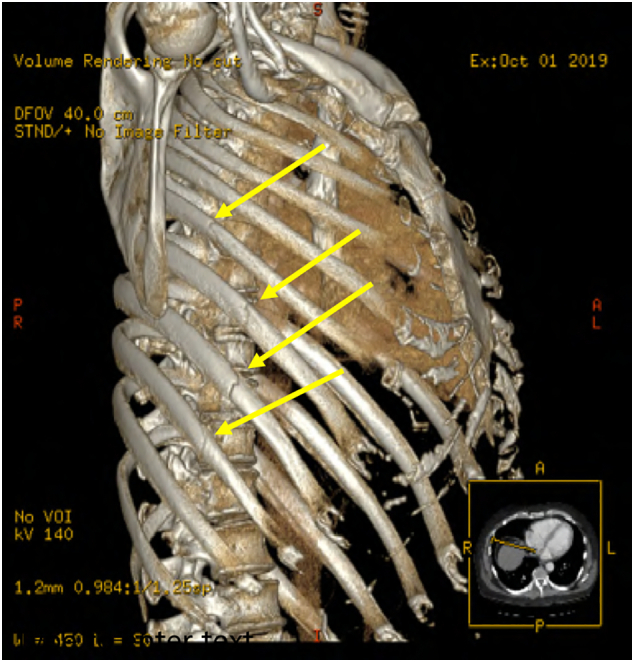
CT reconstructions demonstrating right 6th–9th rib fractures (arrows).

A 48-year-old male was taking out the trash from his home when he lost his footing and fell on his right side resulting in significant pain and shortness of breath. Upon arrival to our institution, he was found to have displaced right 6th through 9th rib fractures without pneumothorax or pulmonary contusion (Fig. 1). Physical exam revealed significant tenderness to palpation over the right chest wall as well as a “clicking” sensation upon deep inspiration. His breathing pattern was rapid and shallow due to severe pain, and his oxygenation saturation was 90% on room air. Initial incentive spirometry measurements were recorded at 500 milliliters (mL), which is only 16% of his predicted value.

The patient was admitted to the intensive care unit (ICU) for pain control and pulmonary hygiene. On hospital days 1 and 2 he continued to experience significant pain despite being given an aggressive multi-modal pain regimen. His incentive spirometer measurements barely improved and he continued to have marginal oxygen saturations requiring oxygen supplementation. On hospital day 4, he was found to be severely tachypneic, hypoxic, and tachycardic, requiring BiPAP support to maintain adequate oxygenation. Pulmonary embolus was ruled out, and he had an epidural placed for better pain control (he initially refused upon admission). Incentive spirometry measurements were still only 32% of his predicted value. Given the slow progression of recovery and the amount of pain the patient was experiencing, the decision was made to proceed with surgical stabilization of the rib fractures. Initially, he was hesitant to undergo surgery but given the amount of pain he was experiencing the continued respiratory compromise, he eventually decided to undergo SSRF on hospital day 8. He was deemed an excellent candidate for stabilization using a minimally invasive intrathoracic approach. After an extensive discussion with the patient regarding the risks and benefits of surgical fixation, he consented to the procedure.

The patient was taken to the operating room for right sided SSRF of ribs 6 through 9. He was intubated with a dual-lumen endotracheal tube, placed in the left lateral decubitus position and prepped and draped in the usual sterile fashion. Initially, a small incision (4 cm) was made in the middle of the palpable rib fractures for exposure from the outside. Next, the pleural space was entered through a 12-millimeter (mm) counter incision in the 8th intercostal space posteriorly. The right lung endotracheal tube portion was then clamped allowing the right lung to collapse for better visualization of the fractures from the inside. Holes were then drilled through non-fractured rib at least 1 cm on either side of the fracture site to allow for rubber guide tubes to be passed through from the inside of the chest cavity outward. The fixation plates were then inserted into the chest via the 12 mm thoracoscope incision and guided to the fractures via cables fed through the rubber guide tubes. Using the cables attached to the plate, we were able to adequately reduce each fracture. The plates were fixated from the outside using bolts and nuts, allowing for both anterior and posterior cortical engagement. Of note, the bolts are fixated to the intrathoracic plates but are able to fold into the plate, allowing for ease of placement into the chest through the 12 mm incision. The bolts are what is attached to the cables which are then pulled through the previously drilled holes, allowing for the nut and washer to secure to the external portion of the rib.

**Fig. 2:**
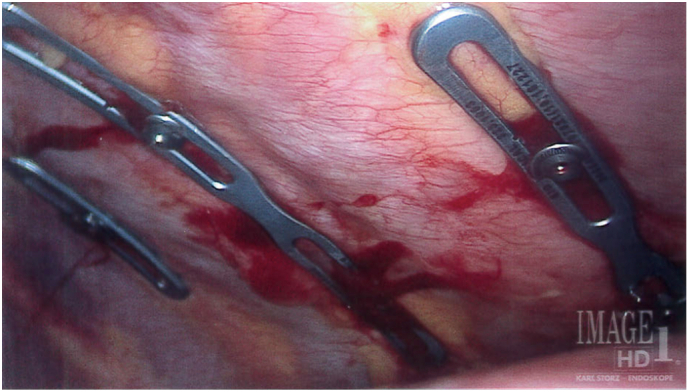
Intrathoracic plates with bicortical fixation and adequate reduction of rib fractures.

**Fig. 3:**
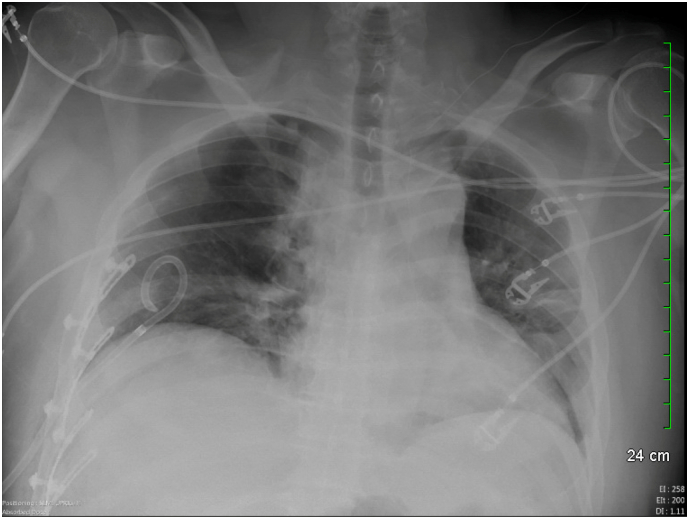
Chest X-ray showing intrathoracic rib plates on right ribs 6 through 9.

Once all four ribs were reduced appropriately and plated, the right chest was much more stable (Fig. 2). The chest cavity was then irrigated thoracoscopically and a 14 French pigtail catheter was placed for further drainage. Cryoablation of the concomitant intercostal nerves was also performed for further pain management. The incisions were copiously irrigated and closed in layers. The patient was extubated and returned to the ICU in a stable condition. A post-operative chest x-ray was obtained and demonstrated in Fig. 3.

**Fig. 4:**
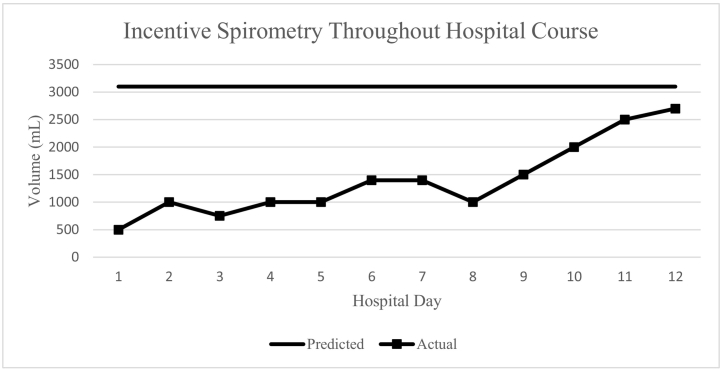
Incentive spirometry use during hospital course. SSRF was performed on Hospital Day 8.

Postoperatively, the patient resumed working with respiratory therapy on an aggressive pulmonary hygiene regimen. He immediately expressed his satisfaction that he could no longer feel his ribs “clicking” on inhalation and that he was pleased with his decision to undergo fixation. On post-operative day (POD) 1, he was out of bed and ambulating the halls, something he had not desired much since admission. His incentive spirometry measurements and oxygenation requirements began to improve, and they improved each day throughout the remainder of his hospital stay. On POD 2, the pigtail catheter was removed without issue and he continued to ambulate even more. By POD 3, his incentive spirometry measurements were 81% of his expected amount and his breathing mechanics had greatly improved (Fig. 4). He no longer required the use of supplemental oxygen was deemed fit for discharge. He was discharged home with support without any issues or complications. Eleven days later, he was seen in clinic and reported a significant improvement in pain and was happy with the progress he had made so far. He was actually requesting to be released back to work at this time, which he was granted.

The patient did follow-up one additional time in the clinic 3 months after his surgery. At that time, his chest pain had completely resolved, he denied any clicking or popping of his rib previous rib fractures and he had no shortness of breath, all of which suggested his ribs had healed. Furthermore, he was back to full work capacity and was sleeping well. Given his significant improvement, no additional imaging was necessary and he was instructed to follow-up in the clinic as needed.

## Discussion

Rib fractures can be some of the most significant injuries in trauma patients with an exponential relationship existing between the number of rib fractures and morbidity and mortality [1,2]. Furthermore, it is estimated that patients suffering rib fractures lose about 70 days of work or normal activity annually [3]. The pulmonary complications that can occur with rib fractures include pneumonia, pneumothorax, acute respiratory distress syndrome, pulmonary embolism, and empyema [5]. It has also been demonstrated that rib fractures cause an increased need for intubation/mechanical ventilation, ICU length of stay, and use of epidural or regional anesthesia (which was the case with our patient) [5]. When a patient sustains rib fractures, the associated pain causes poor breathing mechanics and impaired ventilation. This can subsequently lead to the development of pneumonia and respiratory distress, which can be present in up to 30% of patients with one or more fractures [6]. This was of significant concern in our patient, especially when he acutely decompensated, requiring BiPAP support. It was our worry that had we continued only a multimodal pain control regimen, he would have had a much worse outcome than described.

Although surgical stabilization of rib fractures has been around for over 50 years, it hasn't been until the last two decades that interest in SSRF has heightened due to a combination of surgical innovation as well as clinical observation that despite advancements in nonoperative care, long-term outcomes of patients with severe chest wall injuries still remains poor [7]. Recent studies have shown that surgical fixation in patients with flail chest is associated with fewer days on mechanical ventilator support and a lower incidence of pneumonia [8,9,10]. There has also been strong evidence to suggest SSRF in patients without flail chest to undergo this procedure to help improve pain and respiratory disability-related quality of life [7].

The conventional method to SSRF includes an extrathoracic, muscle-sparing technique that has allowed for decrease post-operative pain and a relatively low complication rate [7,11,12]. While these techniques have proven themselves effective, there is still tentativeness by patients to undergo this procedure due to the level of invasiveness required for fixation [13]. To address these concerns, there has been a recent push to develop new fixation systems and techniques aimed at making smaller incisions and therefore being less invasive [14,15]. Using video assisted thoracoscopic surgery (VATS) to aid in fixation is the next logical step. There are several theoretical advantages to a thoracoscopic approach to SSRF which include: fracture localization under thoracoscopy is intuitive and accurate, expanding the fracture fixation range; fixation under thoracoscopy can avoid trauma to the intercostal vessels, nerves and thoracic viscera via small incisions with direct intrathoracic visualization thereby reducing complications and expediting post-operative recovery; it avoids scapular distraction and eliminates the touchability of the bone fracture plate to the scapula through medial cortical fixation; it can comprehensively explore and treat concurrent intrathoracic injuries in the same period; and minimally invasive and aesthetic surgery is easy for patients to accept [16,17].

Despite the theoretical benefits, early attempts at thoracoscopic SSRF have been limited by both user inexperience and inadequate instrumentation [15]. Further limitations include difficulties with adequate fracture reduction leading to longer operative time, the potential for hardware becoming dislodged and falling into the thoracic cavity, and plate contouring intrathoracically [15,16]. The concern for plate dislodgement is probably one of the more dreaded complications given the plates are now intrathoracic. If the plates did need to be removed, repositioned or tightened, a repeat VATS would be necessary as well as exploration of the wound where the nuts and washers were placed. The good news is the plates and bolts are all one unit, so a VATS with plate/bolt removal should be relatively easy. The nuts and washer are all external to the thoracic cavity so utilizing the plating system wrench will allow for ease of tightening or loosening the plates.

Here we present a case report for thoracoscopic SSRF. Although not perfect, the technique, instrumentation and plates utilized with this case have addressed many of these aforementioned limitations. To our knowledge, this is the first case report published utilizing this new technique, instrumentation and plates. Given the successful outcome in our patient with early return to work and activities of daily living as well as minimal pain with adequate chest wall stability, we recommend further studies looking into this technique and its overall benefits. Given the newness of this technique, indications and contraindications have yet to be fully established. Based on the authors' experience, however, patients need to be able to tolerate one lung ventilation. Furthermore, fractures that are under the scapula can present more challenging, although not impossible depending on surgeon skill level. Lastly, it would not be ideal to utilize this technique for fractures near the costochondral junction when drilling through costal cartilage is required. Overall, the authors would recommend first attempts at this technique be on rib fractures that are on the lateral aspect of the chest wall without additional structures in the way of the surgical field. As SSRF continues to gain popularity and provide positive outcomes for patients with rib fractures, so too will this technique continue to advance.
